# Supplementary material for: Complexome profiling of the Chlamydomonas psb28 mutant reveals TEF5 as an early PSII assembly factor
Source: Plant Cell. 2025 Mar 17;37(6):koaf055. doi: 10.1093/plcell/koaf055 (PMC12164590; doi:10.1093/plcell/koaf055)
Supplement: koaf055_Supplementary_Data [file koaf055_supplementary_data.zip › psb28_interactive.zip!/plots/intensity/qualityPlots/peptides/PDH1_Cre16.g677026.t1.1.html]

### Cre16.g677026.t1.1

Trivial name: PDH1  
SeqCoverage [median]: 15.60  
Mass: 38.15 kDa  
  
 

| Sequence | MissedCleavage | Mass [Da] | wtIntensity | muIntensity | Score |
| --- | --- | --- | --- | --- | --- |
| AAEQLAK | 0 | 729.40 | 0.00 | 2558.83 | 168.74 |
| DALNSALDEELAR | 0 | 1415.69 | 0.00 | 1572.95 | 70.81 |
| HVTLVSFSK | 0 | 1016.57 | 0.00 | 4663.33 | 171.53 |
| SIKPLDRDTLLASVK | 2 | 1654.96 | 0.00 | 2715.49 | 27.77 |
| VLAPYDSEDAR | 0 | 1234.58 | 751.02 | 10141.47 | 92.06 |
|  |  |  | 751.0 | 21652.1 |  |
